# Hybrid Cryogels with Superabsorbent Properties as Promising Materials for Penicillin G Retention

**DOI:** 10.3390/gels9060443

**Published:** 2023-05-26

**Authors:** Marinela Victoria Dumitru, Teodor Sandu, Andreea Miron, Anamaria Zaharia, Ionuț Cristian Radu, Ana-Mihaela Gavrilă, Andrei Sârbu, Horia Iovu, Anita-Laura Chiriac, Tanța Verona Iordache

**Affiliations:** 1 National Institute for Research & Development in Chemistry and Petrochemistry-ICECHIM, 202 Splaiul Independenței, 060021 Bucharest, Romania; dumitrumarinela9@gmail.com (M.V.D.); teodor.sandu@icechim.ro (T.S.); andreea.miron@icechim.ro (A.M.); anamaria.zaharia@icechim.ro (A.Z.); ana.gavrila@icechim.ro (A.-M.G.); andrei.sarbu@icechim.ro (A.S.); 2 Faculty of Chemical Engineering and Biotechnology, University POLITEHNICA of Bucharest, 1-7 Gheorghe Polizu Street, 011061 Bucharest, Romania; radu.ionucristian@gmail.com (I.C.R.); horia.iovu@upb.ro (H.I.)

**Keywords:** hybrid cryogels, biopolymers, kaolin organophilization, stability, penicillin G retention

## Abstract

This present study describes the investigation of new promising hybrid cryogels able to retain high amounts of antibiotics, specifically penicillin G, using chitosan or chitosan–biocellulose blends along with a naturally occurring clay, i.e., kaolin. In order to evaluate and optimize the stability of cryogels, three types of chitosan were used in this study, as follows: (i) commercial chitosan; (ii) chitosan prepared in the laboratory from commercial chitin; and (iii) chitosan prepared in the laboratory from shrimp shells. Biocellulose and kaolin, previously functionalized with an organosilane, were also investigated in terms of their potential to improve the stability of cryogels during prolonged submergence under water. The organophilization and incorporation of the clay into the polymer matrix were confirmed by different characterization techniques (such as FTIR, TGA, SEM), while their stability in time underwater was investigated by swelling measurements. As final proof of their superabsorbent behavior, the cryogels were tested for antibiotic adsorption in batch experiments, in which case cryogels based on chitosan extracted from shrimp shells seem to exhibit excellent adsorption properties for penicillin G.

## 1. Introduction

Nowadays the world is facing major issues related to the contamination of water sources (rivers, lakes, and groundwaters), mainly due to agricultural and industrial practices, with a significant impact on animals, humans, and the environment. In this context, it is recognized worldwide that antibiotics are a concerning class of water contaminants, which affect human life and also the environment [1,2,3,4,5]. Although antibiotics are powerful drugs that have been used for decades in both humans and animals to treat infectious diseases, their presence was observed in tap water, which is alarming due to the toxicity brought upon aquatic organisms and the development of antibiotic-resistant bacterial strains [6,7,8,9,10,11,12].

The presence of antibiotics in dairy products is also of concern, given that they can cause hypersensitive allergic reactions. For this reason, the use of antibiotics in veterinary medicine has been restricted or even banned in some countries. Continued use of antibiotics produces significant residues, which reach directly or indirectly into aquatic and terrestrial environments, leading to antibiotics being labeled as toxic and dangerous chemicals. Thereby, before discharging wastewater into the environment, it is essential to remove antibiotic residues through advanced oxidation processes, converting antibiotic molecules to be less harmful or even completely mineralizing them. However, these processes are costly, and the total removal on an industrial scale is rather challenging. In this regard, it becomes necessary to identify cost-effective and environmentally friendly methods for antibiotic removal [13,14,15,16]. 

Conventional analytical methods, such as mass spectrometry and other advanced methods (microfiltration, ultrafiltration, reverse osmosis, capillary electrophoresis, and electrochemical techniques) have been used to detect and retain pollutants [17,18,19]. However, they all suffer from several drawbacks, primarily related to material costs and time consumption [20]. 

Recently, cryogels have been studied for their ability to be used in separation processes due to their microporous structure with interconnected pores (relatively low back pressure); moreover, their very low densities are beneficial not only for the transport process but also for handling. Cryogels can also be used for various biomedical purposes, of which the most important are tissue engineering and drug delivery, etc. [21,22,23,24,25]. 

Cryogels are polymeric materials, prepared by cryotropic gelification, using either polymers or monomers (hydrophilic or hydrophobic) as precursors, the actual formation being the result of freezing-lyophilization stages [26,27,28,29,30]. These materials not only have the advantages of simplicity and cost-effectiveness of the production process but are also suitable for separation processes as a result of their interconnected macroporous structures, as well as possessing moderate mechanical and chemical resistance [31]. Another advantage of cryogels is given by the use of biopolymer raw materials. The preparation of cryogels based on natural polymers can lead to lower costs, shorter preparation times, and, of course, more biofriendly materials [32]. In this context, several natural polymers, such as biocellulose, chitosan, and alginate, were used to develop these types of materials [33]; thus, they represent an optimal solution for developing promising adsorbent materials for antibiotics as well. 

Chitosan is a natural polymer extracted in large quantities from aquatic resources (shrimp shells, crab shells) and other insect exoskeletons, at relatively low costs [34,35,36]. Chitosan promotes wound healing and also shows bacteriostatic activity. It was observed that by mixing chitosan with a synthetic polymer such as polyvinyl alcohol (PVA), a stiffening effect is produced, which improves the mechanical properties of the obtained materials and is also beneficial for PVA, increasing its solubility in water [37]. Nevertheless, the trends in developing new biofriendly materials target the use of biopolymer blends, such as chitosan–cellulose, chitosan–alginate, and so on [38]. For instance, biocellulose (BC), or bacterial cellulose, is a biomaterial that offers superior properties such as crystallinity, purity, and high mechanical strength. It is biodegradable, ultra-fine, and contains biocompatible nanofibers. BC is generally used for biomedical applications in the production of soft tissues, artificial blood vessels, and artificial skin but also in the production of materials with controlled-release properties [39,40,41,42]. 

In the last decades, researchers have also paid close attention to the use of kaolin (K)-based minerals, because they are cheaper than other minerals, they can act as an active excipient in solid and semi-solid pharmaceuticals, and they can control the effectiveness of dosage forms, which leads to improved bioavailability of the drug [43,44,45,46,47,48]. Among the remarkable properties of K, it is worth mentioning its chemical, thermal, and morphological stability and also some physical properties, such as crystallinity, porosity, specific surface area, particle size, and lack of abrasiveness [47,48,49,50,51]. The major drawback of K is its lower surface area, related to problematic exfoliation procedures, as compared to other minerals such as montmorillonite. However, scientists have found additional strategies for increasing the active surface area of kaolin, in terms of chemical surface modification [45,48,49]. Nevertheless, other issues that need solving are encountered when using natural clays in the preparation of materials. Hence, ensuring the interactions between the clay and the biopolymers remains a challenge every time [43,50,51]. For instance, K and chitosan or BC present low compatibility and, implicitly, limited interactions [43]. For this reason, in this study, the clay underwent an organophilization process, with the aim of creating good biocompatibility with the employed biopolymers. 

**Figure 1:**
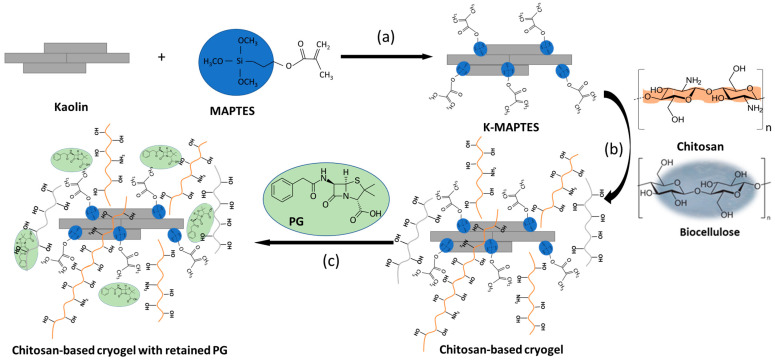
Silylation of K using MAPTES (**a**); proposed interaction mechanism of K-MAPTES with chitosan and biocellulose pendant groups (**b**); and proposed retention mechanism of PG in the biopolymer-based cryogels (**c**).

Thereby, in this work, hybrid cryogels with superabsorbent properties for antibiotics, specifically penicillin G (PG) [52], were developed using chitosan and organophilized K. In this respect, the preparation of the hybrid cryogels involved two stages, namely: (i) chemical organophilization of K by silylation with γ-methacryloxypropyl trimethoxysilane (MAPTES) (as proposed in Figure 1a); and (ii) physical crosslinking of chitosan using ammonium bicarbonate (Figure 1b), which also acted as a foam promoter after contact with the acetic acid solution, used as the solvent for chitosan. The use of ammonium bicarbonate as foam promoter and physical crosslinker was not reported in the literature so far for preparing biopolymer-based cryogels. Therefore, this present study brings some new elements to the known methodology for preparing biopolymer-based cryogels. Furthermore, this present study also investigates the possibility of improving the stability of cryogels during prolonged submergence in water by using chitosan–cellulose blends.

## 2. Results and Discussion

### 2.1. Synthesis of Hybrid Cryogels

In order to study and optimize the stability of cryogels, three types of chitosan were used: (i) commercial chitosan (CC), in which case the following cryogel series was obtained—P1-K, P2-K, P3-K, and P1-K-BC; (ii) chitosan prepared in the laboratory from commercial chitin (CCH), as described in a former study [53], in which case the following cryogel series was obtained—P4-K, P5-K, P6-K, and P4-K-BC; and (iii) chitosan prepared in the laboratory from shrimp shells (CSH), as described in a former study [53], in which case the following cryogel series was obtained—P7-K, P8-K, P9-K, and P7-K-BC. The latter type of chitosan for shrimp shells is expected to contribute more to the stability of cryogels due to the content of native calcium carbonate in its structure. As described in the study of Miron et al. [53], the raw shrimp shells were not demineralized before deproteination and deacetylation and, hence, calcium-carbonate-enriched chitosan was obtained. For each cryogel series with chitosan, additional samples with BC (noted P1-K-BC, P4-K-BC, P7-K-BC) were also prepared and tested. BC was prepared in the laboratory following a fermentation process according to Frone et al. [54]. PG was chosen as the antibiotic model due to its intensive use since its discovery in 1941 in human and veterinary medicine, and in the food industry as a preservative, and also due to its high hydrophilicity [55] and potential to interact with the chitosan-biocellulose-based structure of the prepared cryogels (as proposed in Figure 1c).

#### 2.1.1. Fourier Transform Infrared Spectrometry (FTIR)

**Figure 2:**
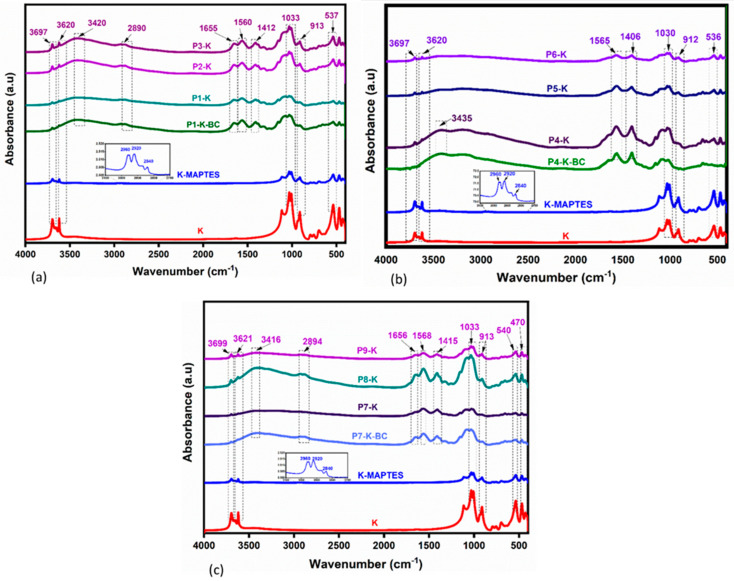
FTIR spectra for hybrid cryogels (**a**) series P1-K, P1-K-BC, P2-K, P3-K; (**b**) series P4-K, P4-K-BC, P5-K, P6-K; (**c**) series P7-K, P7-K-BC, P8-K, P9-K, compared to K and modified K-MAPTES (with the main bands highlighted in medallion).

The FTIR spectra for hybrid cryogels were prepared using different amounts of K, and other types of chitosan were very similar (Figure 2a—series with CC; Figure 2b—series with CCH; Figure 2c—series with CSH). K, the inorganic component, has been organically modified in order to render it biocompatible with the organic component (chitosan). In all spectra of cryogels, the bands corresponding to the stretching vibration of the C=O, N–H, O–H, C–O, and Si–O–Al bonds (specific to the K) can be observed at 1655 cm^−1^, 1560 cm^−1^, 1406 cm^−1^, 1030 cm^−1^, and 912 cm^−1^, respectively. The specific band of O–H stretching vibration in K was observed at 3697 cm^−1^ and 3699 cm^−1^. Figure 2a–c (in medallion of K-MAPTES) also indicates that the clay was successfully organophilized based on the appearance of characteristic bands for C–H (MAPTES) at 2960, 2920, 2880, and 2840 cm^−1^. Further on, the vibrations for the C–H asymmetric and symmetric stretching registered between 2924 and 2872 cm^−1^ in the cryogels spectra are characteristic of chitosan; these bands overlap with those of MAPTES but are more intense. The cryogel samples containing BC exhibit a slightly larger hump in the region 3000–3600 cm^−1^, compared to the counterparts in the series due to its higher capacity for water retention. As a result of K amount variation, the increase in band intensities characteristic of K can also be noticed, in the series: P9 > P8 > P7 (Figure 2c), P6 > P5 > P4 (Figure 2b), P3 > P2 > P1 (Figure 2a), indicating a good homogeneity of resulted cryogels [56,57].

#### 2.1.2. Thermogravimetric Analysis (TGA/DTG)

**Figure 3:**
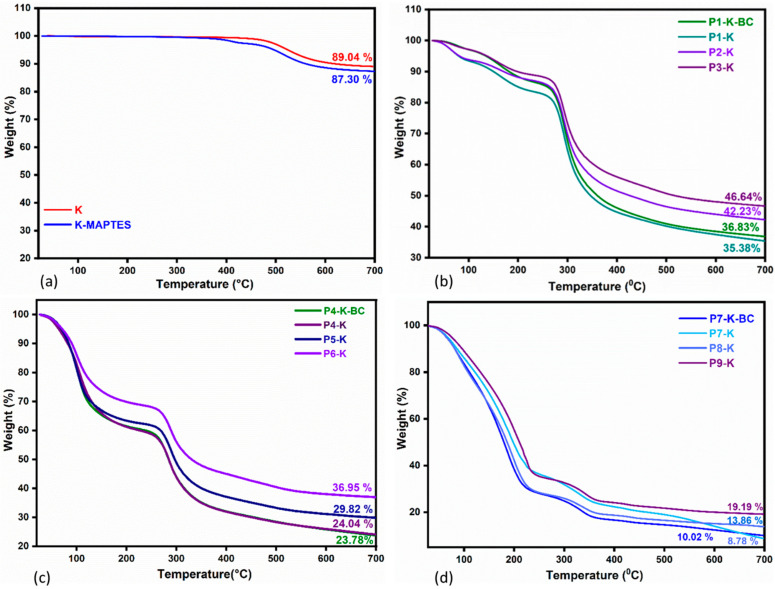
Thermal degradation of K and modified K-MAPTES (**a**) compared to the hybrid cryogels (**b**) series P1-K, P1-K-BC, P2-K, P3-K; (**c**) series P4-K, P4-K-BC, P5-K, P6-K; (**d**) series P7-K, P7-K-BC, P8-K, P9-K.

**Figure 4:**
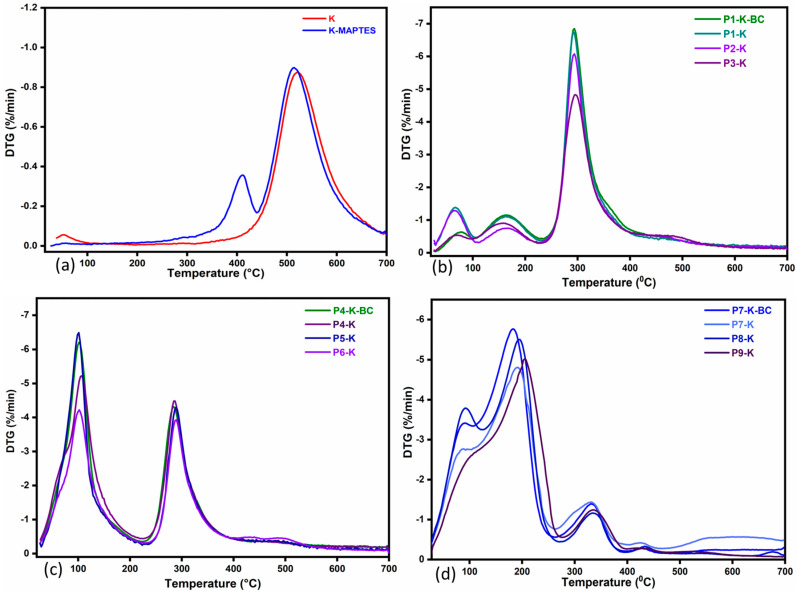
Derivative curves for K and modified K-MAPTES degradation (**a**) compared to the hybrid cryogels degradation (**b**) series P1-K, P1-K-BC, P2-K, P3-K; (**c**) series P4-K, P4-K-BC, P5-K, P6-K; (**d**) series P7-K, P7-K-BC, P8-K, P9-K.

TG analysis was used to investigate the thermal behavior of hybrid cryogels based on natural polymers and clays. The values for maximum decomposition temperatures and mass loss registered for each degradation step are given conveniently in Table S2 (Supplementary Materials) for each cryogel and for the reference K and K-MAPTES. The residual mass of the references and of the hybrid cryogels, respectively, can also be observed in Figure 3a–d. In support of TGA, the derivative curves for sample degradation are given in Figure 4a–d.

Figure 3a shows the mass losses registered for the reference samples K and K-MAPTES. Being an inorganic compound, the clay begins to degrade at very high temperatures, ranging from 450 to 550 °C. In this temperature range, there was a shallow mass loss of only 11%, which can be attributed to water loss as a result of kaolin conversion into metakaolin [43]. This phenomenon can be labeled as a characteristic one for the hybrid cryogels, as well, since it was registered in all the recorded thermograms. For K-MAPTES, the total mass loss of 13% was partly assigned to the thermal decomposition of organic moieties from MAPTES (O-CH-CH_2_, -CH-CH_2_-CH_2_, COO), which takes place around 400 °C. By analyzing the thermal degradation of the hybrid cryogels (Figure 2b–d), for which a larger amount of K-MAPTES was used (i.e., P9-K, P6-K, and P3-K), it can be observed that higher residues were obtained. This observation is also in agreement with the FTIR results, in which case, the same samples registered higher intensities for the characteristic bands of K. Cryogels containing BC are not significantly different from the rest of the samples, which means that BC does not induce essential changes from the thermal stability viewpoint. Interestingly, the mass loss of samples for the three analyzed series increases as a function of the chitosan type, as follows: series P7–P9 with CSH (91–80%) > series P4–P6 with CCH (74–63%) > series P1–P3 with CC (65–53%). Coupled with the DTG data in Figure 4, the advanced mass loss of the cryogel series with CCH and CSH vs. the series with CC may be attributed to the intramolecular water loss and loss of residual acetic acid. 

Therefore, for all the cryogels, the first mass loss around 100–150 °C was specifically attributed to the loss of water and residual acetic acid. The following mass loss, between 250 and 400 °C, was most likely specific to the degradation of chitosan and BC. A particular shift of the maximum degradation temperature (T2) towards higher values was registered for the cryogels from the series with CSH, around 335 °C, vs. 295 and 289 °C maximums recorded for the series with CC and CCH, respectively. The increase in stability for CSH-based cryogels may be attributed to the remanent minerals found in the CSH, as reported by Miron et al. [53]. Subsequently, a slight mass loss was observed for all cryogels between 400 and 550 °C, which indicated that K-MAPTES decomposes according to the mechanism described previously. Thereby, the TGA/DTG results show that the incorporation of K-MAPTES into the chitosan matrix was successfully achieved, leading to the improved thermal stability of the hybrid cryogels; the effect being more intense for samples based on CSH (i.e., P9-K).

#### 2.1.3. Scanning Electron Microscopy (SEM)

**Figure 5:**
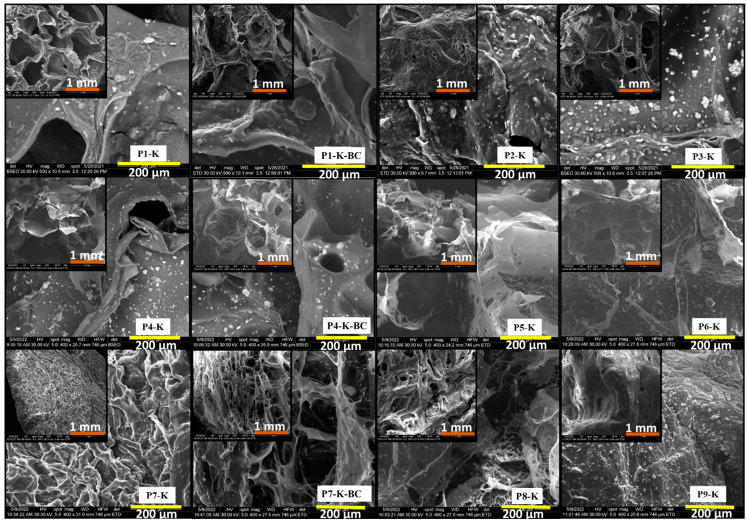
SEM images for lyophilized hybrid cryogels at 1 mm (medallion) and 200 µm scale, P1-K, P1-K-BC, P2-K, P3-K series (**top**), P4-K, P4-K-BC, P5-K, P6-K series (**middle**), and P7-K, P7-K-BC, P8-K, P9-K series (**bottom**).

In Figure 5, the macroporous structures of cryogels as recorded by SEM can be observed for the series with CC (P1-K, P1-K-BC, P2-K, P3-K), with CCH (P4-K, P4-K-BC, P5-K, P6-K), and with CSH (P7-K, P7-K-BC, P8-K P9-K). The clay (K-MAPTES) was homogeneously incorporated into the chitosan or chitosan-BC matrix regardless of the contained amount. The micrograph also revealed that, as the amount of K-MAPTES increases in the three analyzed series, the pores of cryogels diminish, creating smoother interstitial spaces. Furthermore, comparing the cryogels from the point of view of the used chitosan type, an interesting fibrillated microstructure was formed for the series with CSH, especially for P7-K containing the lowest amount of K-MAPTES. It can also be noted that the presence of BC in the cryogels structure did not bring any significant modifications to the cryogels morphology.

#### 2.1.4. Nitrogen Intrusion Porosimetry, Brunauer–Emmett–Teller (BET)

N_2_ adsorption–desorption isotherms, relying on the principles of Brunauer, Emmett, and Teller’s (BET) theory for multilayer adsorption of gas molecules on solids, allowed assessing the specific BET surfaces, pore volume (Vp), a specific surface area of pores (S_BET_), pore surface area (A_p_), and average pore diameter (D_p_), of all the cryogels series and reference counterparts (K-MAPTES, K) (values summarized for convenience in Table 1).

The cryogels prepared by the cryogenic method showed similarities in textural parameters with the reference K and K-MAPTES. For instance, the specific surfaces and the pore volume of cryogels, with a higher K-MAPTES content (P3-K, P6-K, and P9-K) are significantly higher compared to those containing a lower amount of K-MAPTES, regardless of the type of chitosan used, which means that all the textural parameters are closely related to the amount of K incorporated. It can also be noted that the cryogels based on BC present improved BET surfaces, pore surface, diameter, and volume, compared to the same cryogels without BC, which may be due to their fibrillated texture. 

N_2_ adsorption and desorption isotherms of the reference K, K-MAPTES, and the three cryogel series (as given in Figures S1–S4, Supplementary Materials) presented type H4 hysteresis curves, characteristic to mesoporous structures according to the IUPAC classification [57]. This type of isotherm is registered for mesoporous adsorbents such as oxide gels and adsorbents, with cylindrical pores which determine the formation of a hysteresis upon gas desorption [58]. Pore size distribution curves were determined using the desorption data. In this respect, the hybrid cryogels presented four representative peaks, which suggested the presence of mesopores with diameters between 3.169 and 4.152 nm, while for the reference K and K-MAPTES, only three representative peaks were observed, with most mesopores having a diameter of 28.5 nm. 

#### 2.1.5. Determination of the Swelling Degrees (*SD*s)

**Figure 6:**
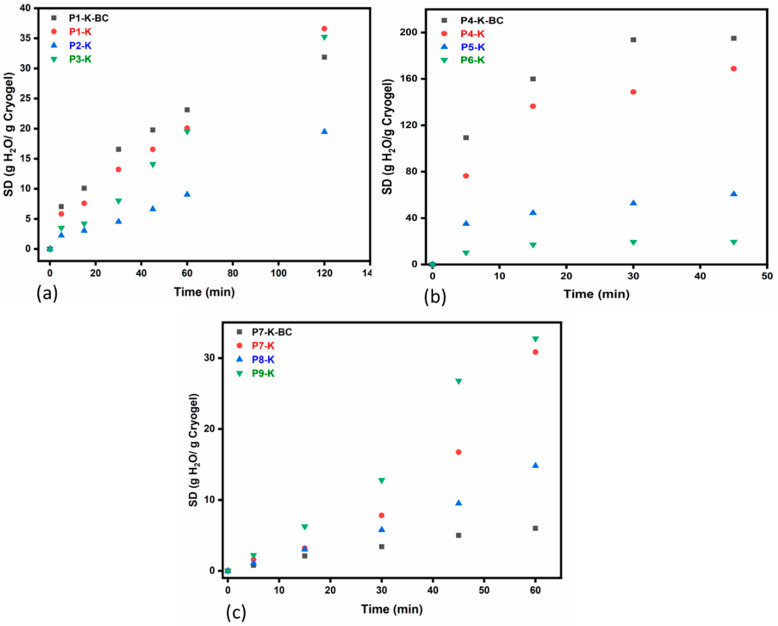
Variation in swelling degrees in time for the cryogel series based on CC (**a**), CCH (**b**), and CSH (**c**), performed in distilled water at room temperature.

The variation in *SD*s in time for the cryogel samples, shown in Figure 6 (and summarized in Table S3, Supplementary Materials) has indicated that, after 1–2 h at pH 5.5 (the pH of distilled water), cryogels begin to fragment requiring timely monitoring of the swelling process. Following this study, it was found that the stability in time underwater for the three series of cryogels is rather low compared to other superabsorbent chemically crosslinked gels prepared with the same chitosan types [59], in which study, stability at 24 h was reported. The highest stability in water was achieved in this study by the CC-based hybrid cryogels (up to 2 h), followed by the series with CSH (up to 1 h), and, finally, the CCH-based cryogels started to fragment after only 45 min. The low water stability of the CCH series was also influenced by the very high and fast water adsorption, in which case the highest *SD* values were recorded (up to 195 g H_2_O/g Cryogel, Figure 6b). Yet, the results are somehow explainable because physical chitosan gels are known to present lower mechanical properties vs. chemical gels [60]. Nevertheless, the *SD*s reported in this present study were comparable with those presented by Neblea et al. [59], with the difference that high *SD*s values were achieved herein in a very short time, 1–2 h of swelling, which may be due to the fact that they were not chemically crosslinked. 

It is also noteworthy the fact that the increase in K-MAPTES had a particularly favorable effect on the swelling degree only for the series of cryogels with CC and CCH (Figure 6a,b). In these cases, the samples with higher K-MAPTES content, P3-K, and P6-K, seem to present moderate water adsorption in time compared to the samples with lower amounts of K-MAPTES, i.e., P1-K and P4-K, respectively. For these series as well, BC leads to even higher water adsorption which eventually accelerates the fragmentation process of cryogels. On the other hand, for the series based on CSH (Figure 6c), BC is strongly affecting the swelling capacity of cryogels, which may not be necessarily a disadvantage if this action prevents the fragmentation of cryogels without diminishing the capacity for subsequent PG adsorption. In this ultimate series, P7-K seems to present the highest *SD*s, which is again justified by the low amounts of K-MAPTES used. 

Following these results, it can be noted that the addition of K-MAPTES can lead to an increase in stability for the chitosan physical gels at pH values of 5.5, in distilled water, due to the very few studies that report measurements of chitosan physical gels beneath 6–7 pH (as result to their pH sensitivity). For instance, Ezati et al. [61] measured the behavior of chitosan films in the 4–10 pH range in order to evaluate the color change. The films were submerged under various buffer solutions only for 10 min.

#### 2.1.6. Evaluation of PG Retention by Batch Adsorption Measurements

**Figure 7:**
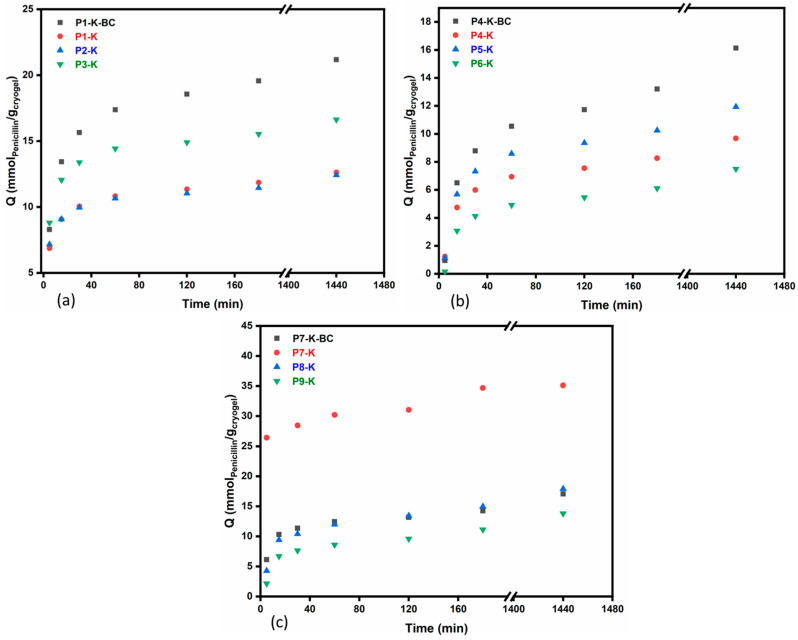
Profiles of PG retention in time for the cryogel series based on CC (**a**), CCH (**b**), and CSH (**c**), performed in distilled water at room temperature.

For all the cryogels series the adsorption capacity was studied using the target antibiotic PG. Its high solubility in water made the study less difficult. However, due to the PG tendency to decompose quickly in aqueous solutions, forming penicilloic acid in just 24 h, as demonstrated by Wang et al. [62], the decrease in PG in supernatants was evaluated versus the absorbance registered for the 0.02 mol/L PG solution freshly measured at each time interval. Evaluating the profiles of PG retention in Figure 7 (values briefly given for all the cryogels in Table S4 in mmol/g and Table S5 in mg/g, Supplementary Materials), it can be noticed there is a slight resemblance with those recorded for the *SD* with the difference that the stability of all cryogels was this time higher (up to 24 h). In this respect, it should be noted that PG addition increases the pH of the solution from 5.5 (distilled water) to 6.5, which prevents the chitosan structure to relax and afterward fragment. Chitosan, by nature, is a biopolymer that solubilizes in weakly acidic aqueous media (pH < 6.5), therefore, its relaxation and subsequent solubilization are influenced by the pH of the solution [63]. This is why the cryogels were far more stable in this set of measurements compared to the *SD* evaluation. For the series with CC and CCH (Figure 7a,b), the cryogels with BC, meaning P1-K-BC and P4-K-BC, exhibit the highest adsorption capacities, of 21.1 and 16.1 mmol/g, respectively, after 24 h. It can also be observed that the series with CCH is less efficient for retaining PG, thus confirming the results obtained previously for *SD*. 

Further on, the retention profiles for cryogels in the series CSH also follow a similar variation to the ones observed for the *SD*s. In this series, P7-K recorded the highest value of adsorption capacity (35.1 mmol/g), while the lowest value was attained by the sample containing the highest amount of K-MAPTES, i.e., P9-K (13.8 mmol/g). Nevertheless, all three series of cryogels showed massive improvements in adsorption capacity for PG compared to similar studies. For instance, Nourmoradi et al. [64] obtained a maximum adsorption capacity for PG of 88.5 mg/g over 60 min using modified montmorillonite, while Vakili et al. [65] reported a retention capacity of PG for chitosan-based adsorbents near 2165 mg/g. In order to demonstrate the fast and quantitative retention of PG by the newly prepared cryogels, a comparison can be made starting with the lowest adsorption capacity from the three series at 1 h, of P6-K, in which case an adsorption capacity of 4.9 mmol/g (meaning 1750 mg/g) was measured. If the highest adsorption capacity is evaluated, for sample P7-K at 1 h, it results in a value of 30.1 mmol/g (meaning 10,742 mg/g). Therefore, the results show that cryogels based on CSH and K-MAPTES can adsorb up to 5-fold more PG in 1 h compared to other adsorbents recently described in the literature.

In Table 2, the parameters obtained by fitting the adsorption data into a second-order kinetic model proposed by Ho and McKay [66], given in (Equation (3)), are also in agreement with the porosity measurements. According to the literature, diagrams based on plotting t/q(t) against t present a linear relationship [67] (data given in Supplementary Materials Figures S5–S7). Except for P4-K-BC, the data fits almost perfectly to the selected model, and the fact that all the R^2^ are above 0.999 means that the model is properly describing the investigated dependency [67].

## 3. Conclusions

The study provided information on the synthesis of hybrid cryogels suitable for retaining antibiotics found in wastewater; biopolymers being used in conjunction with natural clays for this purpose. The three types of chitosan (CC, CCH, and CSH) were used along with BC and modified K out of a desire to develop stable hybrid cryogels for prolonged submergence under water. However, the swelling study showed relatively low stability in time for all the cryogels but revealed high water adsorption capacities in the first hour. In agreement with the *SD* measurements, the batch evaluation of the cryogels for PG adsorption revealed that cryogels based on CSH and K-MAPTES can retain 10,742 mg of PG/g of cryogel in the first hour. Therefore, it can also be presumed that the physical crosslinking of cryogels can lead to improved adsorption capacities for PG. The kinetic study revealed a pseudo-second-order mechanism of PG adsorption for all the analyzed cryogels, almost perfectly fitted to the model of Ho and McKay. In addition, the stability of cryogels was found to be greatly influenced by the pH of the solutions. Thus, at a pH near 6.5, the cryogels maintain their integrity for up to 24 h. 

FTIR spectroscopy and TG analysis confirmed that K-MAPTES was successfully incorporated into the biopolymer matrix and that CSH-based cryogels decompose at higher temperatures compared to the other cryogel series based on CC and CCH. SEM micrographs, together with BET analysis, provided information regarding the surface morphology and porosity of prepared cryogels, indicating that higher K-MAPTES amounts lead to smoother surface morphologies but higher pore surface areas, and higher pore dimensions and volumes. However, coupled with the batch adsorption results, it was noticed that the smoother surface morphology of cryogels slows down or limits PG adsorption. 

Concludingly, the results obtained in this study reveal that the hybrid cryogels developed from chitosan and modified kaolin feature remarkable retention capacities for PG and present significant potential for use as adsorbent materials for PG retention in wastewater. 

## 4. Materials and Methods

### 4.1. Materials

Natural Kaolin (K, Acros Organics) and the silane coupling agent, γ-methacryloxypropyltrimethoxysilane (MAPTES, by Sigma Aldrich, 98% purity), were used without further purification. In order to obtain the three cryogels series, the following types of chitosan were used: (i) commercial chitosan (CC, ≥75% deacetylation degree, Mn = 2.056 × 10^5^ g/mol, supplied by Sigma Aldrich and used as received); (ii) chitosan from commercial chitin (CCH, 77% deacetylation degree, Mn = 4.7291 × 10^5^ g/mol) prepared according to Miron et al. [53]; and (iii) chitosan from shrimp shells (CSH, 76% deacetylation degree, Mn = 9.058 × 10^3^ g/mol), prepared according to Miron et al. [53]. Pure demineralized water (Millipore) was used to solubilize the chitosan. Biocellulose (BC) was kindly provided by our collaborator from the University “Politehnica” of Bucharest in the form of a swollen membrane as described by Frone et al. [54]. Penicillin G (PG, purity ≥ 100%, M = 356 g/mol), acetic acid (99%), and ammonium bicarbonate (NH_4_HCO_3_, 99.5%) were purchased from Sigma-Aldrich. 

### 4.2. Synthesis of Hybrid Cryogels Based on Chitosan, Biocellulose, and Kaolin

#### 4.2.1. Modification of K with MAPTES Coupling Agent

The K modification process was performed by silylation with MAPTES, by combining two procedures described in the literature [48,49]. The appropriate amount of K was dried at 70 °C for 6 h before the synthesis. In a typical trial, 1 g of dried K and 5 mL of MAPTES (wt. ratio of 1:5) were mixed under magnetic stirring for 1 h. The mixture is further refluxed at 110 °C, for 24 h, under an inert atmosphere of nitrogen gas. The final suspension was separated by centrifugation and washed first with toluene (30 mL) at a gravimetric ratio of solid: liquid about 1:30, and afterward with water (30 mL), for 30 min at 6000 rpm. The obtained solid fraction was dried at 90 °C for 24 h, and a mortar was used to reduce the particle size. 

#### 4.2.2. Preparation of Hybrid Cryogels 

The preparation of the three series of hybrid cryogels involved several steps. For each trial, a glass beacon of 25 mL was used to solubilize 0.3 g of chitosan in 12 mL of acetic acid solution (2% concentration was used for commercial chitosan and chitosan from commercial chitin, while for the chitosan from shrimp shells, a 50% concentration was needed), at gravimetric ratio between chitosan: solvent mixture of 1:40, under magnetic stirring (room temperature, 3 h). For samples noted P1-K-BC, P4-K-BC, and P7-K-BC, 0.6 g of BC was added at a wt. ratio chitosan:BC of 1:2, after complete dissolution of chitosan. The organophilized K was added in different wt. ratios in the homogenized solution of chitosan, i.e., chitosan:K-MAPTES = 6:1 for samples (P1-K, P4-K, P7-K, P1-K-BC, P4-K-BC, P7-K-BC), 3:1 for samples (P2-K, P5-K, P8-K), and 2:1 for samples (P3-K, P6-K, P9-K). When a homogeneous distribution was reached, the crosslinking/foaming agent (NH_4_HCO_3_) was added in a wt. ratio of chitosan:NH_4_HCO_3_ = 1:2. The mixture was homogenized by energic stirring with a spatula, until the foaming of the chitosan solution was complete, followed by immediate freezing at −20 °C. The samples were completely frozen just after 120 h. Subsequently, the samples were fresh cut to approximately 1 cm^3^ and lyophilized at −55 °C for 72 h.

It should also be noted that a high concentration of acetic acid was required for the dissolution of chitosan prepared from shrimp shells (CSH), and, therefore, a supplemental washing step was needed for samples P7-K-BC, P8-K, and P9-K, prior to freezing and lyophilization. Each sample was washed in dialysis bags and soaked in 50 mL of water (4 cycles, 1 h per cycle), using a shaker, at a rotational speed of 200 rpm, at 22 °C. The wastewater obtained after each washing cycle was collected and evaluated by UV–Vis spectrometry. A peak around 280 nm, specific for very high concentrations of acetic acid [68], occurred in all the spectra of waste waters, collected after 1 h. Yet, the concentration of acetic acid decreased significantly after the 4th washing cycle.

### 4.3. Characterization Techniques

The FTIR spectra were recorded using a ThermoScientific Summit Pro spectrophotometer, performing 16 scans for each sample at a resolution of 4 cm^−1^, in the spectral range 4000–400 cm^−1^. The samples were analyzed as potassium bromide pellets.

The TGA curves of the obtained cryogels were recorded using the TA Q500 instrument. Each sample was heated from 25 to 800 °C, at a heating rate of 10 °C/min, under an inert atmosphere of nitrogen gas.

SEM images were recorded using a Quanta Inspect F Scanning Electron Microscope (Waltham, MA, USA) equipped with an emission gun, and a 1.2 resolution field (EGF). Aiming at confirming the success of clay incorporation within the polymer matrix, SEM images were recorded for all the cryogel series. The samples were placed on a carbon strip, which was further placed on a copper grid. The samples were coated (metalized) for 30 s with a thin layer of gold.

Nitrogen intrusion porosimetry (Brunauer–Emmett–Teller—BET) was performed using a NOVA 2200 Quantachrome Instruments porosimeter, with the end of assessing the specific surface and micropore size. Prior to the measurements, the cryogels were milled. The actual analysis by the BET method involved the adsorption/desorption of nitrogen after the samples have been degassed for 4 h, at 40 °C.

### 4.4. Determination of Swelling Degrees (SD)

In order to determine the stability of cryogels under prolonged submerge under water, the swelling degree (*SD*) of the cryogels (P1-K ÷ P9-K) was studied for 2 h; this being the limit after which the cryogels fragment. The samples were soaked in 10 mL of water, in Falcon tubes, with a capacity of 50 mL; the stirring was ensured by a MultiTherm shaker device (Cool-Heat-Shake) Benchmark (200 rpm, 22 °C). *SD*s were calculated at different time intervals and were assessed according to Equation (1), whereas *m_S_* (g) and *m_d_* (g) represent the weight of the swollen and dried cryogel, respectively.

(1) $$SD=\frac{m_{s}-m_{d}}{m_{d}}$$

### 4.5. Retention Capacity of Cryogels for Penicillin G, as Model Antibiotic

The prepared cryogel samples (P1-K ÷ P9-K) were tested for their capacity to retain PG as the model antibiotic. In this respect, 0.02 mol/L PG solution was prepared by the dissolution of 0.356 g of PG into 50 mL of H_2_O under magnetic stirring (200 rpm), while ensuring light protection. Each cryogel sample (approximately 0.02 g) was contacted with a volume of 10 mL of PG solution. At different time intervals (5, 15, 30, 60, 120, 180, 1440 min), the liquid phase (supernatant) was tested by UV–Vis spectroscopy at λ = 322 nm (specific wavelength of PG) [62] in order to evaluate the retention capacity of cryogels for PG (*q*, mmol PG/g cryogel) as given in Equation (2), where *Ci* (mmol/L) and *Cf* (mmol/L) are the initial and final concentrations of PG in the supernatant, *Vs* (L) and *Mcryogel* (g) represent the volume of the PG solution and the weight of the dried cryogel taken into account. The UV–Vis spectra were recorded using a UV–Vis ThermoScientific EVOLUTION 260 BioSpectrophotometer.

(2) $$q=\left(Ci-Cf\right)*Vs/Mcryogel$$

The adsorption mechanism was analyzed using a pseudo-second-order kinetic model, described by Ho and McKay [66], as presented in Equation (3).

(3) $$\frac{1}{q_{t}}=\frac{t}{q_{e}}+\frac{1}{k_{2}q_{e}^{2}}$$

where *q_e_* and *q_t_* are the adsorption capacity (mg/g) at equilibrium and at time *t* (min), respectively, and *k*_2_ (g mg^−1^ min^−1^) is the pseudo-second-order adsorption rate constant. 

## Figures and Tables

**Table 1:** BET-specific parameters for the three cryogel series compared to K and K-MAPTES.

Samples	Surface Area BET (m^2^ g^−1^)	Pore Surface Area (BJH) (m^2^ g^−1^)	Pore Diameter for Desorption (BJH) (nm)	Pore Volume (BJH) (Measured la P/P0 = 0.99) (cm^3^ g^−1^)
K-MAPTES	7.373	9.216	28.750	0.034
K	9.703	10.010	3.169	0.024
P1-K	1.812	3.253	3.627	0.005
P1-K-BC	2.310	4.279	4.152	0.006
P2-K	3.705	6.177	4.152	0.010
P3-K	3.067	11.400	4.152	0.013
P4-K	1.318	2.273	4.752	0.004
P4-K-BC	2.213	3.068	4.543	0.005
P5-K	2.851	3.212	4.543	0.007
P6-K	3.980	5.053	4.543	0.010
P7-K	1.720	2.310	3.315	0.005
P7-K-BC	1.967	5.068	3.627	0.008
P8-K	6.484	9.480	3.969	0.018
P9-K	8.322	11.300	4.152	0.023

**Table 2:** Parameters fitting for PG adsorption according to a pseudo-second-order kinetic model.

Sample	k_2_ (g mg^−1^ min^−1^)	q_e_ (mg/g) *	R^2^
P1-K-BC	5.37 × 10^−6^	7537	0.9999
P1-K	1.23 × 10^−5^	4491	0.9999
P2-K	1.17 × 10^−5^	4423	0.9999
P3-K	9.31 × 10^−6^	5918	0.9999
P4-K-BC	2.04 × 10^−6^	5742	0.9985
P4-K	5.34 × 10^−6^	3445	0.9998
P5-K	3.99 × 10^−6^	4247	0.9996
P6-K	2.66 × 10^−5^	2669	0.9995
P7-K-BC	3.83 × 10^−6^	6077	0.9995
P7-K	5.77 × 10^−6^	13,362	0.9999
P8-K	2.93 × 10^−6^	6378	0.9996
P9-K	2.98 × 10^−6^	4915	0.9993

* For this assessment, the adsorption capacity at 24 h was used as equilibrium adsorption capacity.

## Data Availability

The data presented in this study are available on request from the corresponding author. The data are not publicly available due to ongoing studies.

## Supplementary Materials

The following supporting information can be downloaded at: https://www.mdpi.com/article/10.3390/gels9060443/s1, Figure S1. Adsorption–desorption isotherms for K-MAPTES and K references; Figure S2. Adsorption–desorption isotherms for cryogels series with commercial chitosan P1-K, P1-K-BC, P2-K, and P3-K; Figure S3. Adsorption–desorption isotherms for cryogels series with chitosan obtained from commercial chitin P4-K, P4-K-BC, P5-K, and P6-K; Figure S4. Adsorption–desorption isotherms for cryogels series with chitosan obtained from shrimp shells P7-K, P7-K-BC, P8-K, and P9-K; Figure S5. Fitting results to the pseudo-second-order for cryogels series with commercial chitosan P1-K, P1-K-BC, P2-K, and P3-K; Figure S6. Fitting results to the pseudo-second-order for cryogels series with commercial chitosan P4-K, P4-K-BC, P5-K, and P6-K; Figure S7. Fitting results to the pseudo-second-order for cryogels series with commercial chitosan P7-K, P7-K-BC, P7-K, and P7-K; Table S1. FTIR spectral assignment of bands for each cryogel series; Table S2. Maximum decomposition temperatures and mass losses in each decomposition step as evaluated from TG analysis, for the three cryogel series and of K and K-MAPTES; Table S3. Swelling degrees of the three cryogel series performed in distilled water; Table S4. Adsorption capacities of cryogels for PG [mmol/g], from 0.02 mol/L solution, in batch mode. Table S5. Adsorption capacities of cryogels for PG [mg/g], from 0.02 mol/L solution, in batch mode.
