# Supplementary material for: Hybrid Cryogels with Superabsorbent Properties as Promising Materials for Penicillin G Retention
Source: Gels. 2023 May 26;9(6):443. doi: 10.3390/gels9060443 (PMC10297641; doi:10.3390/gels9060443)
Supplement: Supplementary file 1 [file gels-09-00443-s001.zip › gels-2398609-supplementary.pdf]

Hybrid cryogels with superabsorbent properties as promising materials for penicillin G retention

Marinela Victoria Dumitru[1], Teodor Sandu[1], Andreea Miron[1], Anamaria Zaharia[1], Ionut Cristian Radu[2], Ana-Mihaela Gavrila[1], Andrei Sarbu[1], Horia Iovu[2], Anita-Laura Chiriac[1*], Tanța Verona Iordache[1*]

[1]National Institute for Research&Development in Chemistry and Petrochemistry-ICECHIM, 202 Spl. Independenței, 060021 Bucharest, Romania

[2]University POLITEHNICA of Bucharest, Faculty of Chemical Engineering and Biotechnology, 1-7 Ghe. Polizu Street, 011061 Bucharest, Romania

Table S1. FTIR spectral assignment of bands for each cryogel series

Sample Series	Wavenumbers (cm^{-1})	Spectral assignments
**P1-K P1-K-BC P2-K P3-K**	3697-3420	O-H from hydroxyl group (moisture)
	2890	C-H stretching vibrations from MAPTES structure
	1655	C=O stretching of amide 1
	1560	N-H bending of amide II
	1033	C-O stretching
	913	Si-O-Al (Kaolin)
	537	Si-O-Si
**P4-K P4-K-BC P5-K P6-K**	3697-3435	O-H from hydroxyl group (moisture)
	1565	C=O stretching of amide 1
	1406	N-H bending of amide II
	1030	C-O stretching
	912	Si-O-Al (Kaolin)
	536	Si-O-Si
**P7-K P7-K-BC P8-K P9-K**	3699-3416	O-H from hydroxyl group (moisture)
	2894	C-H stretching vibrations MAPTES structure
	1656	C=O stretching of amide 1
	1568	N-H bending of amide II
	1033	C-O stretching
	913	Si-O-Al (Kaolin)
	540	Si-O-Si

Table S2. Maximum decomposition temperatures and mass losses in each decomposition step as evaluated from TG analysis, for the three cryogel series and of K and K-MAPTES

Samples	T_{1Max} ˚C	T_{2Max} ˚C	T_{3Max} ˚C	T_1 %	T_2 %	T_3 %	F%
**K**	-	-	522.3	-	0.51	10.45	10.96
**K-MAPTES**	-	410.8	512.8	-	1.20	11.50	12.70
**P1-K-BC**	165.7	292.7	461.3	10.47	43.40	9.30	63.17
**P1-K**	161.8	292.5	466.8	13.64	41.63	9.35	64.62
**P2-K**	165.9	292.6	466.4	10.95	37.46	9.36	57.77
**P3-K**	158.4	295.5	486.4	9.47	34.42	9.47	53.36
**P4-K-BC**	101.7	284.3	479.2	35.36	32.68	8.18	76.22
**P4-K**	106.0	285.3	464.5	34.68	33.48	7.8	75.96
**P5-K**	100.8	288.7	496.2	33.73	29.25	7.2	70.18
**P6-K**	102.3	289.3	494.5	27.19	27.91	7.95	63.05
**P7-K-BC**	183.7	328.9	432.3	71.41	11.95	6.62	89.98
**P7-K**	192.4	332.2	434.8	62.96	14.71	13.55	91.22
**P8-K**	195.0	334.4	433.4	70.90	10.43	4.81	86.14
**P9-K**	204.4	335.8	442.4	63.49	12.36	4.96	80.81

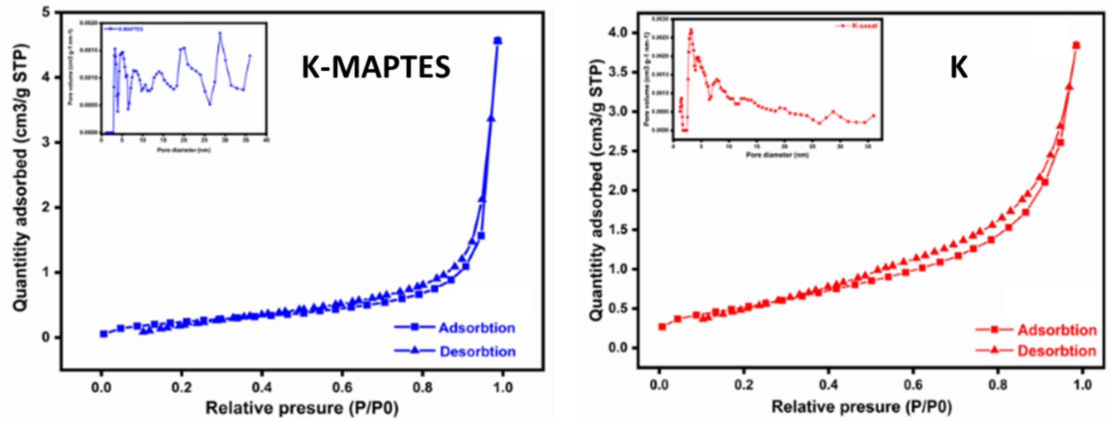

Figure S1. Adsorption-desorption isotherms for K-MAPTES and K references

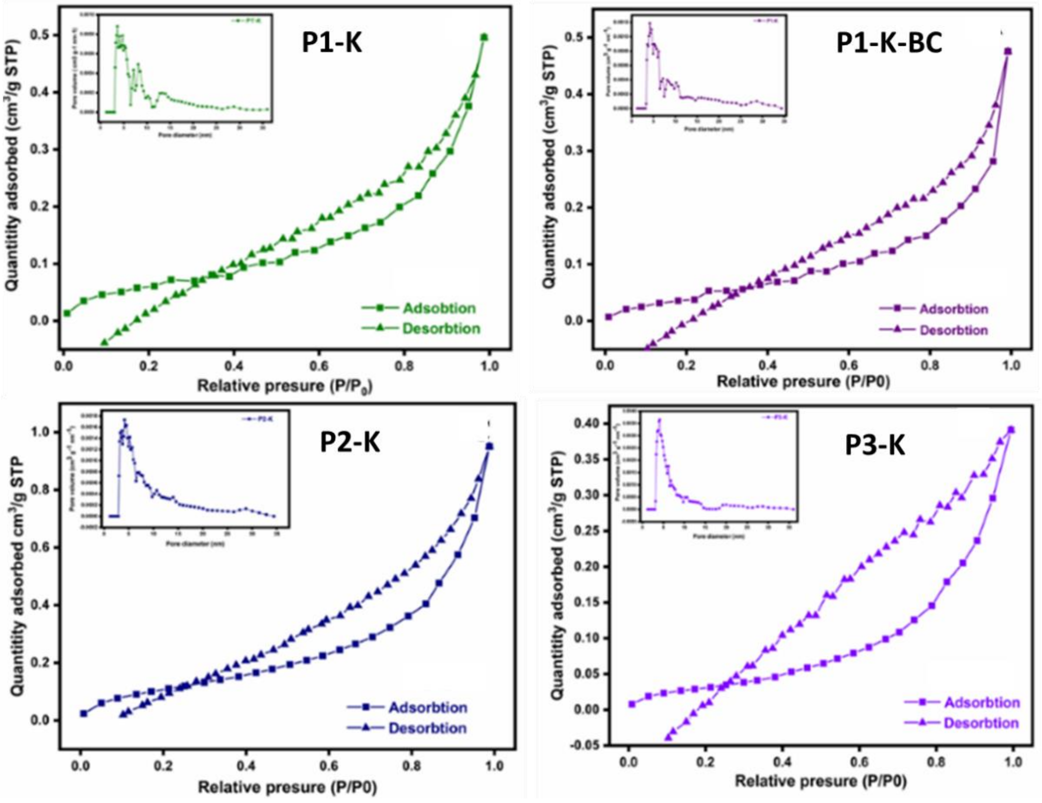

Figure S2. Adsorption-desorption isotherms for cryogels series with commercial chitosan P1-K, P1-K-BC, P2-K, and P3-K

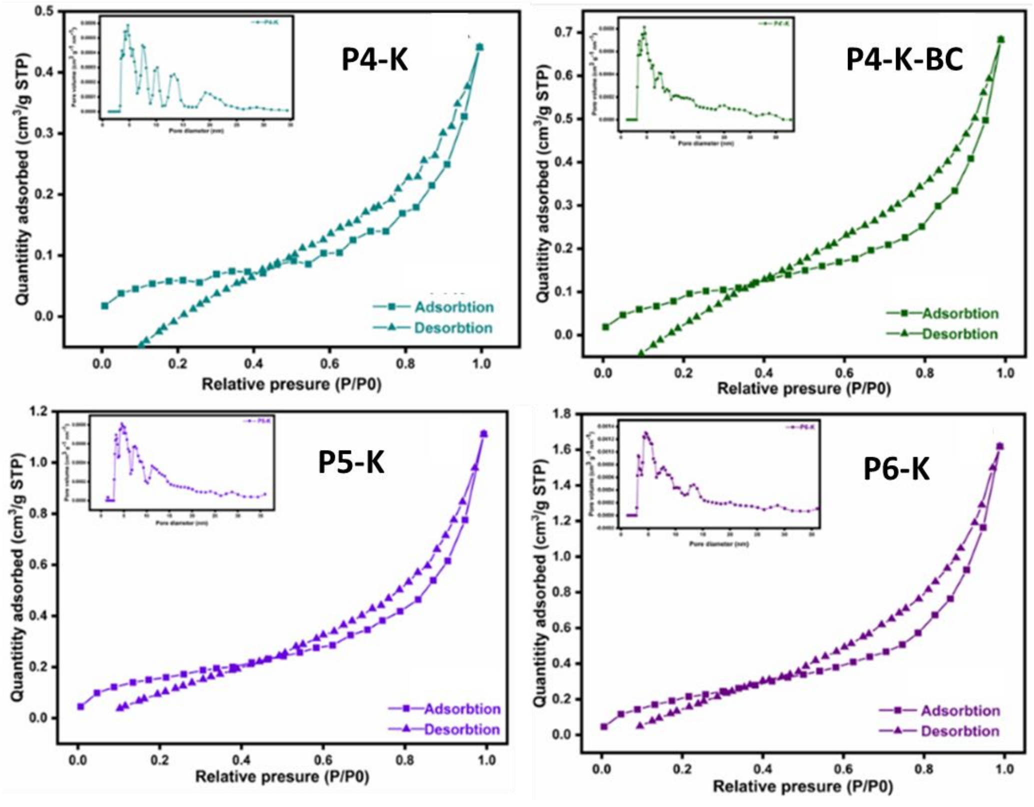

Figure S3. Adsorption-desorption isotherms for cryogels series with chitosan obtained from commercial chitin P4-K, P4-K-BC, P5-K, and P6-K

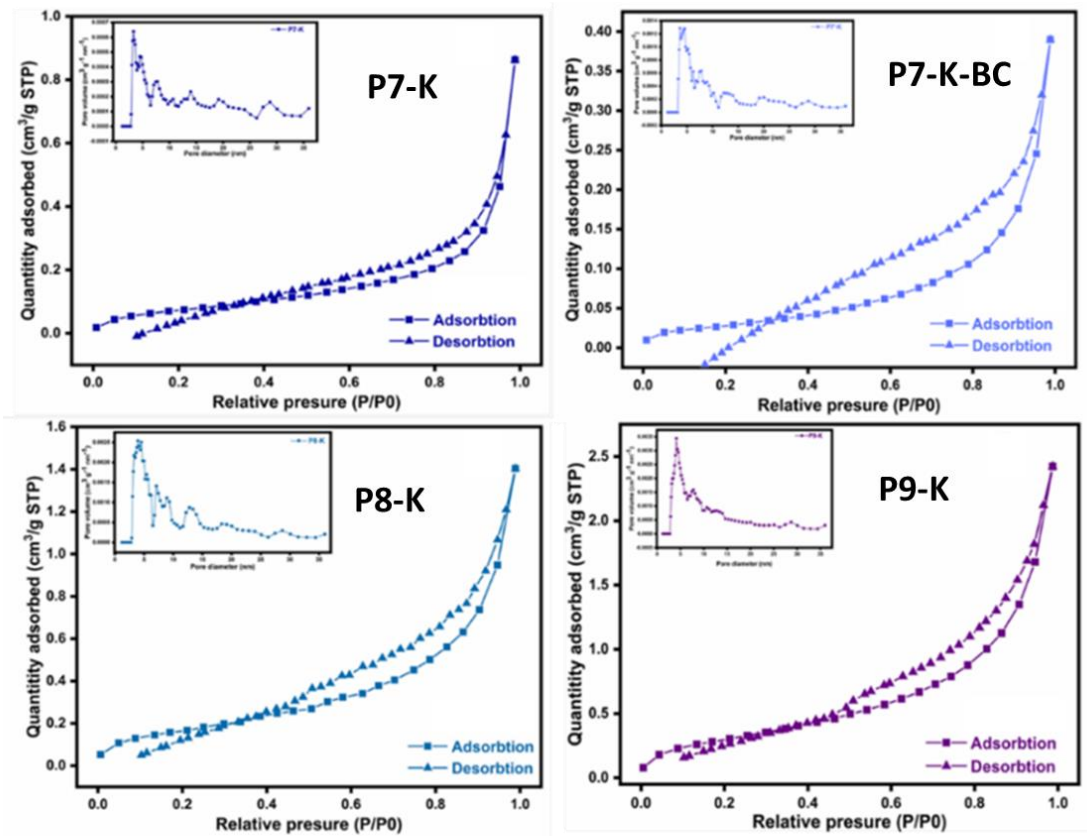

Figure S4. Adsorption-desorption isotherms for cryogels series with chitosan obtained from shrimp shells P7-K, P7-K-BC, P8-K, and P9-K

Table S3. Swelling degrees of the three cryogel series performed in distilled water

**Time (min)**	**SD (g H_2O/ g Cryogel)**											
	**P1-K-BC**	**P1-K**	**P2-K**	**P3-K**	**P4-K-BC**	**P4-K**	**P5-K**	**P6-K**	**P7-K-BC**	**P7-K**	**P8-K**	**P9-K**
5	7.0	5.8	2.2	3.5	109.3	76.4	35.1	10.2	0.79	1.6	1.1	2.2
15	10.1	7.6	3.0	4.2	159.9	136.4	44.5	17.2	2.11	3.2	3.0	6.3
30	16.6	13.2	4.5	8.0	193.7	148.7	52.8	19.42	3.4	7.8	5.8	12.8
45	19.8	16.5	6.6	14.1	195.0	168.8	60.6	19.62	5.0	16.7	9.5	26.8
60	23.1	20.1	9.0	19.6	-	-	-	-	6.0	30.8	14.8	32.7
120	31.9	36.6	19.5	35.2	-	-	-	-	-	-	-	-

Table S4. Adsorption capacities of cryogels for PG [mmol/g], from 0.02 mol/L solution, in batch mode

Time (min)	Q ($mmol_{PG}/g_{cryogel}$)											
	P1-K-BC	P1-K	P2-K	P3-K	P4-K-BC	P4-K	P5-K	P6-K	P7-K-BC	P7-K	P8-K	P9-K
5	8.3	6.8	7.1	8.8	0.9	1.2	1.1	0.2	6.1	26.4	4.3	2.2
15	13.4	9.1	9.0	12.0	6.5	4.7	5.7	3.0	10.3	37.5	9.4	6.7
30	15.6	10.0	9.9	13.3	8.8	5.9	7.3	4.1	11.4	28.4	10.4	7.6
60	17.3	10.8	10.6	14.4	10.5	6.9	8.5	4.9	12.4	30.1	12.0	8.6
120	18.5	11.3	11.0	14.9	11.7	7.5	9.3	5.4	13.1	31.0	13.4	9.6
180	19.5	11.8	11.4	15.5	13.2	8.2	10.2	6.1	14.2	34.7	14.9	11.1
1440	21.1	12.6	12.4	16.6	16.1	9.6	11.9	7.5	17.0	35.1	17.9	13.8

Table S5. Adsorption capacities of cryogels for PG [mg/g], from 0.02 mol/L solution, in batch mode

Time (min)	Q ($mg_{PG}/g_{cryogel}$)											
	P1-K-BC	P1-K	P2-K	P3-K	P4-K-BC	P4-K	P5-K	P6-K	P7-K-BC	P7-K	P8-K	P9-K
5	2952	2445	2550	3134	337	437	404	57	2187	9409	1523	771
15	4780	3227	3232	4292	2313	1685	2021	1092	3679	13362	3356	2389
30	5569	3575	3548	4765	3127	2132	2607	1466	4045	10131	3712	2725
60	6184	3851	3794	5135	3753	2467	3056	1750	4430	10743	4274	3066
120	6607	4039	3928	5303	4176	2686	3330	1942	4693	11047	4778	3417
180	6967	4220	4075	5529	4701	2942	3649	2174	5080	12339	5328	3966
1440	7537	4491	4423	5918	5742	3445	4247	2669	6077	12491	6378	4915

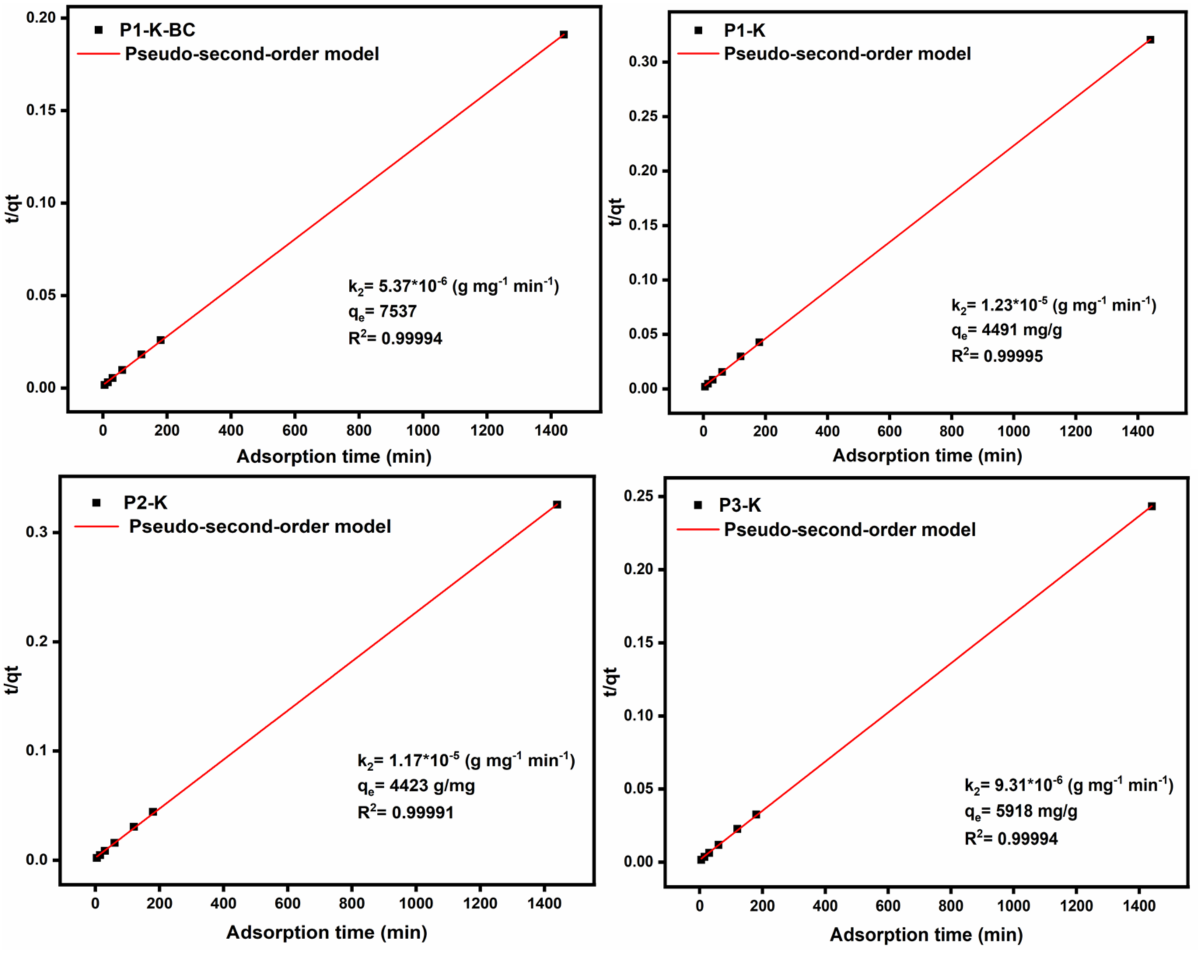

Figure S5. Fitting result to the pseudo-second-order for cryogels series with commercial chitosan P1-K, P1-K-BC, P2-K, and P3-K

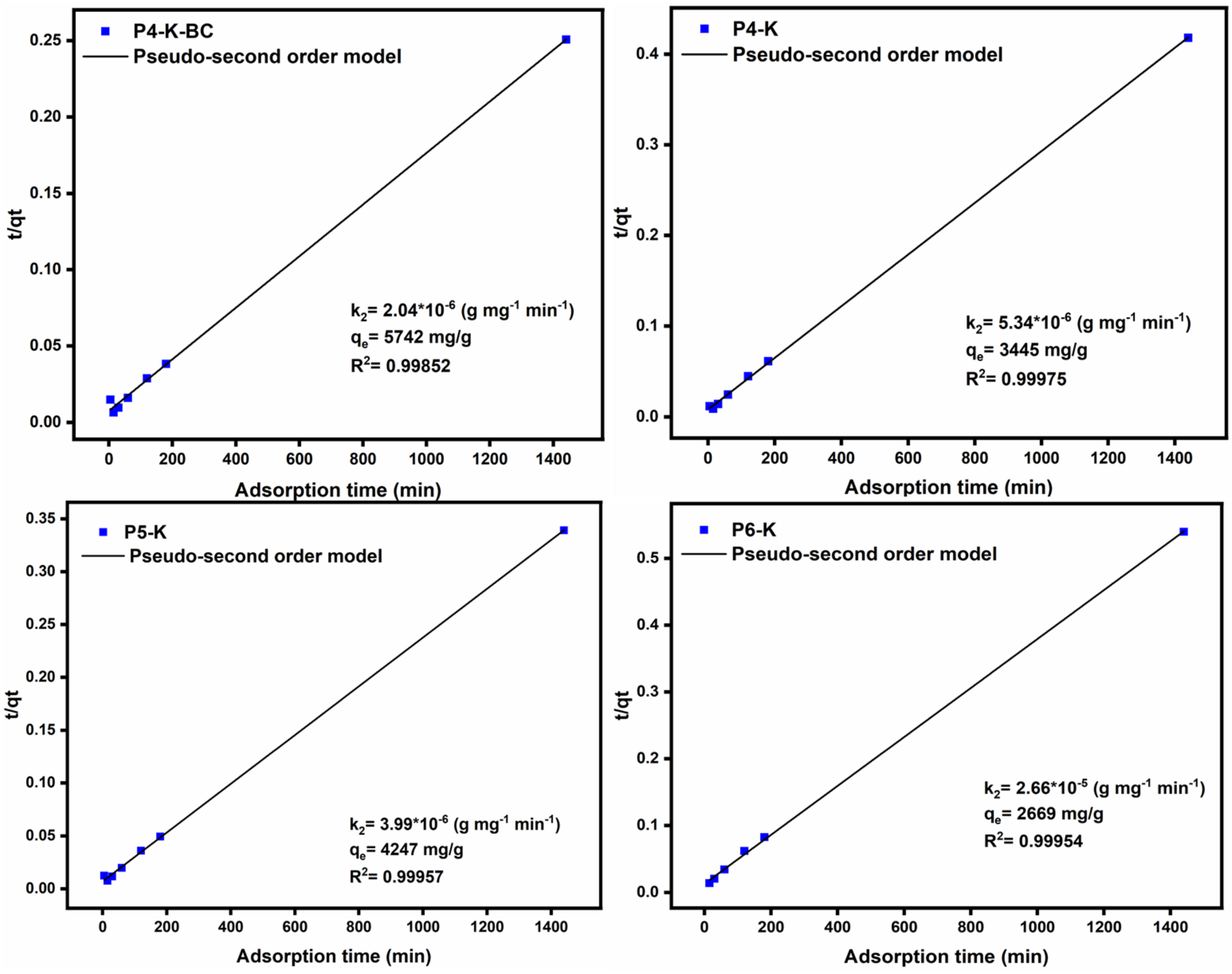

Figure S6. Fitting result to the pseudo-second-order for cryogels series with commercial chitosan P4-K, P4-K-BC, P5-K, and P6-K

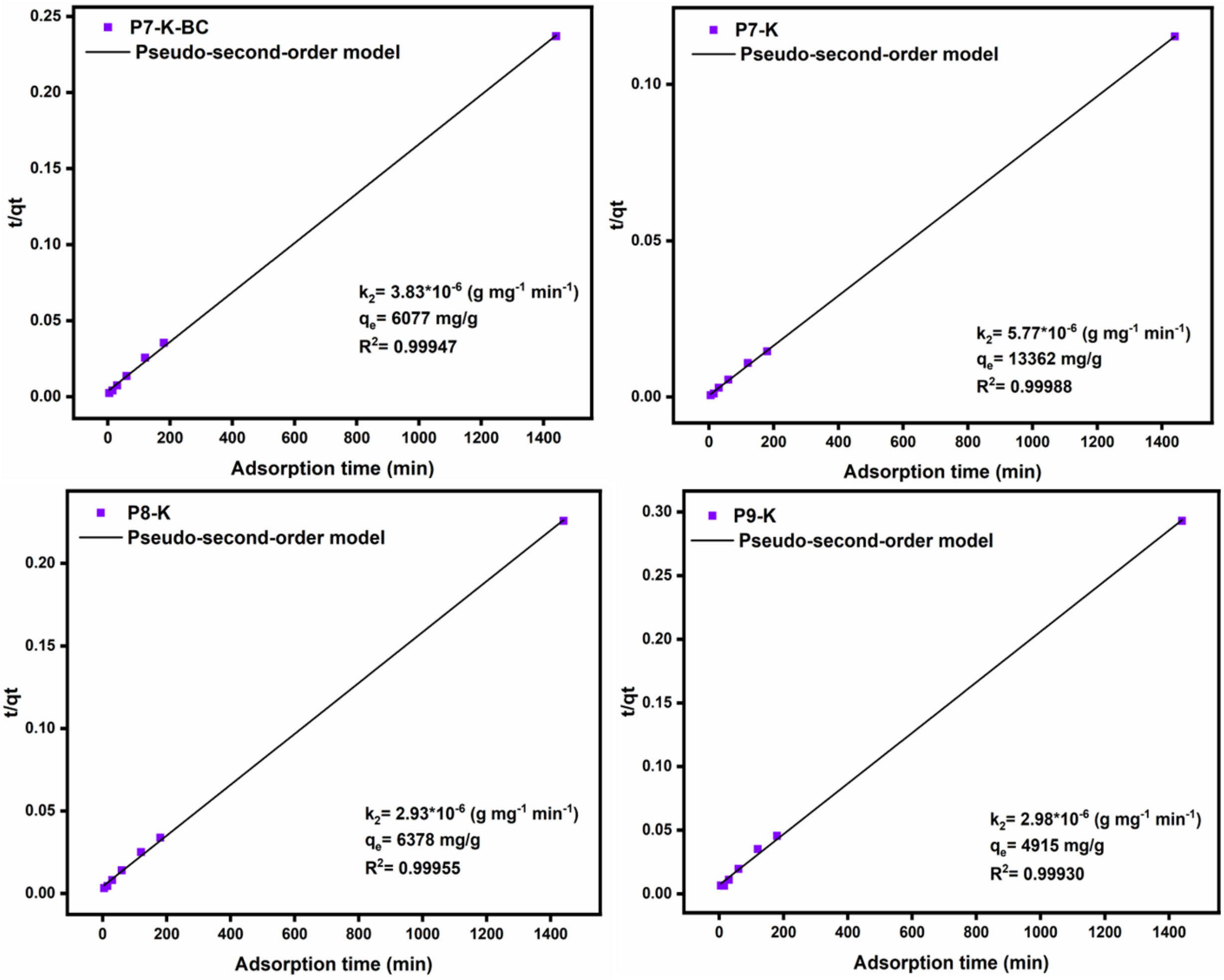

Figure S7. Fitting result to the pseudo-second-order for cryogels series with commercial chitosan P7-K, P7-K-BC, P7-K, and P7-K
